# Methods of postural analysis in connection with the stomatognathic system. A systematic review

**DOI:** 10.25122/jml-2022-0327

**Published:** 2023-04

**Authors:** Cezar Ioniță, Alexandru Eugen Petre, Roxana-Simina Cononov, Anatoli Covaleov, Brindusa Ilinca Mitoiu, Adriana Sarah Nica

**Affiliations:** 1 Department of Occlusion and Fixed Prosthodontics, University of Medicine and Pharmacy Carol Davila, Bucharest, Romania; 2 Department of Restorative Odontotherapy, University of Medicine and Pharmacy Carol Davila, Bucharest, Romania; 3 Department of Rehabilitation, University of Medicine and Pharmacy Carol Davila, Bucharest, Romania

**Keywords:** dental occlusion, posture, trigeminal nerve

## Abstract

This systematic review aimed to identify the main tools used to analyze the relationship between the postural and stomatognathic systems. The study followed the PRISMA guidelines, and data were collected from Science Direct and PubMed databases to identify articles published until December 2022. After applying inclusion and exclusion criteria, 26 articles were selected from the initial 903 articles. The selected articles were full-text studies in English or Romanian, examining the relationship between dental occlusion and posture, measuring postural parameters using various tools, implementing occlusal changes, evaluating patients with permanent dentition, or analyzing the connection between occlusion and posture in a unidirectional manner. The findings indicate that orthognathic surgery and orthodontic mouthguards can significantly enhance postural balance and athletic performance. In addition, 63% of the studies concluded that varying modifications and occlusal conditions impact posture. Notable differences exist concerning posture and Angle dental occlusion classes, and different occlusal devices used to simulate malocclusion can affect patients' postural systems in response to external stimuli. The stabilometry platform is the predominant method for measuring postural parameters; however, other researchers have employed raster stereography, photogrammetry, mobile phone apps, and the Fukuda-Unterberger test. Consequently, interventions targeting the stomatognathic system should consider potential variations in the postural system.

## INTRODUCTION

Postural control, primarily assessed in bipedal static stance, utilizes the center of pressure (CoP) as the main measuring parameter [1]. The CoP is the global ground reaction force vector that accommodates body sway [2]. The force plate is a commonly used instrument to study postural control, allowing subjects to assume different stances, such as open eyes (OE), closed eyes (CE), open mouth (OM), and closed mouth (CM) [3,4].

Posture adjustment is a complex process that involves the integration of inputs from the somatosensory, visual, and vestibular systems at the central nervous system level, where sequences of information are decoded and integrated to achieve posture adjustments [5]. In recent years, many researchers have studied the influence of the oral cavity on body posture by analyzing the stomatognathic system in various stances, such as: placing silicon panels between teeth [6], clenching teeth with maximal force, clenching on cotton rolls, performing laterotrusion to the right until an edge-to-edge bite [7], protrusion and lateral movements [8]. The proprioception of the oral cavity is represented by receptors located in muscles, periodontal ligaments, and the temporomandibular joint, which provide the muscular system with the necessary information to position body segments in space [9].

The first clear description of the relationship between posture and dental occlusion was introduced in the literature by Rocabado et al. in 1982, and this generated a different perspective on the connections between the stomatognathic system and other structures of the human body [9]. Fibers from the periodontal ligaments perform functions similar to those from the joint capsules where the Ruffini corpuscles are found. These pressure receptors enable very fine sensorial stimulations that modify the perception of the stomatognathic system [10]. Gangloff investigated the effects of trigeminal nerve afferents on posture by performing anesthesia at the level of the mandibular nerve [11]. Postural, optical, osteopathic, and occlusal variables are often clinically associated, emphasizing the need for multidisciplinary medical treatment to address these dysfunctions [10].

Posturography, a clinical assessment of postural system function, involves various tests, including mobility, balance, and muscle tone assessment [12]. Static and dynamic tests are performed to assess the posture, with computerized posturography being the most commonly used method. However, some tests, such as Barré's vertical test, finger test, Fukuda test, and craniocorpography do not use computerized posturography [12]. Other postural evaluation methods described in the literature are raster stereography [13], digital spinometria [14], and mobile apps [8].

This systematic review aimed to investigate the relationship between the stomatognathic system and posture and to identify the main tools used for such analysis. The study aimed to answer two primary research questions: (1) What are the primary tools used to evaluate the connection between the postural system and stomatognathic system? and (2) What is the nature of the relationship between the stomatognathic system and posture?

The review utilized a selection of studies that assessed changes in postural parameters before and after interventions that targeted dental occlusion. These studies were selected, analyzed, and compared to provide insight into the relationship between posture and the stomatognathic system.

## Material and methods

### Protocol and registration

This systematic review adheres to the Prisma guidelines for data reporting. An extensive electronic literature search was conducted, focusing on studies published no earlier than 2000. We included only peer-reviewed original articles published in English or Romanian that reported methods for measuring body posture parameters and those that investigated the relationship between posture and modifications in the stomatognathic system.

### Inclusion/exclusion criteria for the selected studies

The following inclusion criteria were applied to select studies: full-text articles published in English or Romanian that analyze the relationship between dental occlusion and posture, studies that present a clinical part with tests and measurements, studies that measure postural parameters using various tools such as stabilometry platforms or mobile phones, studies in which changes are made at the occlusal level, both through different positioning of the mandible and the interposition of objects between the dental arches such as splints or cotton rolls, studies that examine patients with permanent dentition, studies that analyze the relationship between occlusion and posture in one direction. The exclusion criteria included articles not written in English or Romanian, articles studying the posture of edentulous subjects, temporary dentition, mixed dentition, or people over 65 years old.

### Timeline for the search process

**Figure 1:**
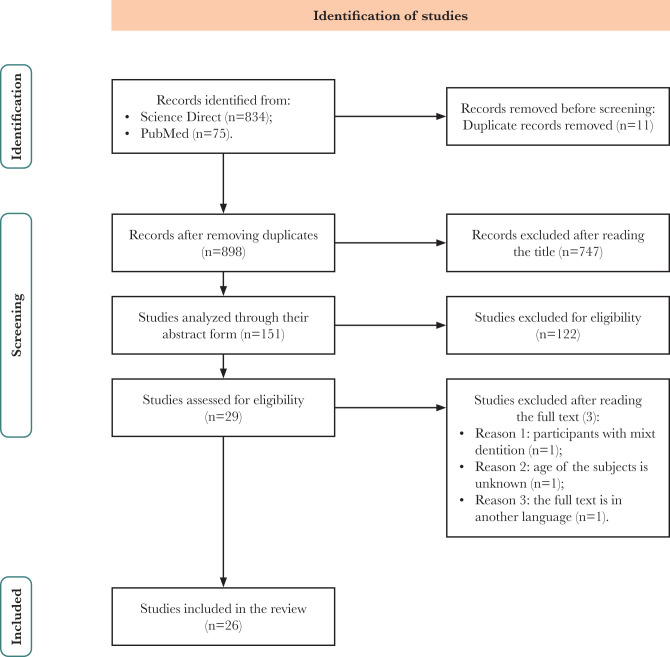
The PRISMA flow diagram illustrating the flow of information through different phases of the systematic review.

Our systematic search yielded 903 studies, comprising 834 and 75 records from Science Direct and PubMed, respectively. After removing duplicates and screening titles, we selected 151 records for full-text review based on predefined inclusion and exclusion criteria. After reviewing the abstract and applying the inclusion and exclusion criteria, 29 articles were kept. After reading the articles thoroughly, 26 results remained. Three studies were excluded due to mixed dentition in subjects, lack of information on subjects' age, or full-text articles not being available in English or Romanian. The selection process is summarized in Figure 1. A detailed summary of the characteristics of the included studies is presented in Table 1.

**Table 1:** Articles included in the review.

Reference	Year	Patients	Men	Women	Tests used
**Sonia Julià-Sánchez *et al*. [5]**	2015	25	15	10	Biodex body balance system (Biodex, NY, USA)
**Milani, Ramin Sharifi *et al*. [15]**	2000	30	22	8	Fukuda-Unterberger Tests
**Julià-Sánchez, Sonia *et al*. [16]**	2016	10	10	-	Biodex body balance system (Biodex, NY, USA)
**Souza, Juliana A. *et al*. [17]**	2014	51	-	-	Photogrammetric analysis + baropodometric evaluation
**Perinetti, Giuseppe *et al*. [18]**	2006	26	13	13	10-Hz sampling frequency vertical force platform (Lizard, Como, Italy)
**Ohlendorf, Daniela *et al*. [6]**	2014	23	5	18	SonoSens Monitor (Gefremed GmbH, Chemnitz, Germany)
**Cuccia, Antonino Marco [19]**	2011	168	60	108	Baropodometric platform
**Baldini, Alberto *et al*. [20]**	2013	44	30	14	Posturostabilometric force platform (Postural Health Station, DL Medica S.p.A. Milano, Italy)
**Leroux, Eric, *et al*. [21]**	2018	7	4	3	Force platform (Cyber-SabotsTM, SABOSOFT software, France)
**Dias, Amândio, *et al*. [22]**	2020	15	15	-	Kinematic analysis, five high-speed cameras (Basler A602fc, Basler Vision Technologies, Ahrensburg, Germany)
**März, Karoline *et al*. [7]**	2017	44	21	23	Rasterstereography (Formetric 4D®, Diers International GmbH, Schlangenbad, Germany
**Iacob, Simona Maria *et al*. [8]**	2020	29	4	25	PostureScreen® Mobile app, version 8.5, developed by Posture Co. Inc. (Trinity, FL, USA)
**Parrini, Simone *et al*. [13]**	2018	30	13	17	Rasterstereography (Formetric 4D®, Diers International GmbH, Schlangenbad, Germany
**Maurer, C. *et al*. [23]**	2021	59	19	41	Plantar pressure plate GP multisens (GeBioM, Münster, Germany)
**Amaricai, Elena *et al*. [24]**	2020	95	29	66	Stabilometric analysis - PoData system (Chinesport, Udine, Italy)
**Maurer-Grubinger, C. *et al*. [25]**	2020	800	393	407	Video raster stereography. back scanner “ABW-BodyMapper” (ABW GmbH, Frickenhausen/ Germany)
**Bracco, P. *et al*. [26]**	2004	95	23	72	Computerized posturographic and stabilometric footboard (Moebius Alpha by Ergomed srl, Cremona, Italy)
**Michalakis, Konstantinos X. *et al*. [27]**	2019	20	14	6	Ppressure assessment system (MatScan, Tekscan Inc., Boston, MA, USA)
**Tardieu, Corinne *et al*. [28]**	2009	10	6	4	Static and dynamic posturography platform Multitest Equilibre apparatus (Framiral, Cannes, France)
**Michelotti, Ambrosina *et al*. [29]**	2006	26	14	12	Stabilometric platform (Lizard, Lemax s.r.l., Como, Italy)
**Tecco, Simona, *et al*. [30]**	2010	30	-	30	Electronic Baropodometer Multi-Sensor system, D.B.I.S. (Digital Biometry Images Scanning) (Diagnostic Support S.r.l. Via Dora 1 - 00198 Roma, Italy)
**Eriksson, Per Olof *et al*. [31]**	2019	54	14	40	Wireless video-based camera system for 3D movement recording
**Dias, Amândio A. *et al*. [32]**	2018	13	13	-	Force platform (Biosignalplux, Lisbon, Portugal)
**Grondin, Francis *et al*. [33]**	2017	22	15	7	iPhone (iPhone 5, Clinometer; Peter Breitling, Version 3.3)
**Nota, Alessandro *et al*. [4]**	2016	44	10	34	Posturostabilometric force platform (Postural Health Station, DL Medica S.p.A. Milano, Italy)

### Literature search strategy and selection process

To identify relevant studies, we conducted a comprehensive search of the Science Direct and PubMed databases from inception to December 2022, using keywords such as "posturology," "dental occlusion," "posture," and "stabilometry platform," both individually and in combination. We screened the resulting articles by reviewing their abstracts and extracted data from those that met our eligibility criteria. If an article was considered potentially eligible, the full text was retrieved and reviewed. The selection process was carried out by a single reviewer.

### Data collection process and risk of bias assessment

After reviewing the articles, we extracted data on the study type, subjects participating, methods and materials, and results. Data extraction was done by a single reviewer. The extracted data were grouped into tables based on the characteristics, materials, and methods used in each study, facilitating the comparison and analysis of the results. The quality of the articles was assessed by checking the selection of the subjects participating in the study, the test conditions, the equipment used, the data collection, and the interpretation of the results (Table 2). Each article was assigned a score based on our assessment, as outlined in Table 3. Based on their scores, the articles were classified into two groups: high-quality and low-quality.

**Table 2:** Methodological reliability scoring for reviewed articles.

**I. Study design (15✓)**
A. Objective: description of measurement or procedure under investigation (✓)
B. Objective: outline what is known from previous studies (✓)
C. Sample characteristics: subjects described (✓)
D. Sample characteristics: assessors described (✓)
E. Sample size: subjects adequate (✓)
F. Sample size: assessors adequate (✓)
G. Sample representation: subjects representative of the population (✓)
H. Sample representation: assessors representative of the population (✓)
I. Sample qualifications/experience: all assessors with necessary experience (✓)
J. Sample subject variability: heterogeneous subjects (✓)
K. Minimization of random error: equipment described (✓)
L. Minimization of random error: subjects described (✓)
M. Minimization of random error: assessors described (✓)
N. Clinically stable subjects: yes (✓)
O. Period of time between measurements: adequate (✓)
**II. Study measurements (4✓)**
P. Measurement method: appropriate to the objective (✓)
Q. Blind measurement: blinding (✓)
R. Reliability: an adequate level of intraobserver agreement (✓)
S. Reliability: an adequate level of interobserver agreement (✓)
**III. Statistical analysis (2✓)**
T. Statistical analysis: appropriate for data (✓)
U. Confounders: confounders included in the analysis (✓)
**IV. Results (2✓)**
V. Meaningfulness (e.g., ICC, SEM, CI, kappa): provided (✓)
W. Generalized to clinical/research context: yes (✓)
**MAXIMUM NUMBER OF ✓s = 23**

**Table 3:** Methodological reliability scores of reviewed articles.

Articles	A	B	C	D	E	F	G	H	I	J	K	L	M	N	O	P	Q	R	S	T	U	v	w	Points	%
**Sonia Julià-Sánchez *et al***. [5]	✓	○	✓	×	○	○	✓	○	×	✓	✓	✓	×	✓	✓	✓	×	○	○	✓	○	✓	✓	15.5	67.4
**Milani, Ramin Sharifi *et al***. [15]	✓	✓	✓	×	○	○	○	○	×	✓	✓	✓	×	✓	✓	○	×	✓	○	✓	✓	✓	✓	16	69.5
**Julià-Sánchez, Sonia *et al***. [16]	✓	✓	✓	×	○	○	×	○	×	×	✓	✓	×	✓	✓	✓	×	✓	○	✓	✓	✓	✓	15	65.2
**Souza, Juliana A. *et al***. [17]	✓	✓	✓	✓	✓	✓	○	✓	○	✓	✓	✓	○	✓	✓	✓	○	✓	○	✓	×	✓	✓	19.5	84.8
**Perinetti, Giuseppe *et al***. [18]	✓	○	✓	×	○	○	○	○	×	✓	✓	✓	×	✓	○	✓	×	✓	○	✓	×	○	○	13.5	58.7
**Ohlendorf, Daniela *et al***. [6]	✓	✓	✓	×	✓	○	✓	○	×	✓	✓	✓	×	✓	✓	✓	×	✓	○	✓	✓	✓	✓	17.5	76.1
**Cuccia, Antonino Marco** [19]	✓	✓	✓	✓	✓	✓	✓	✓	○	✓	✓	✓	○	✓	✓	✓	✓	✓	○	✓	✓	✓	✓	21.5	93.5
**Baldini, Alberto *et al***. [20]	✓	✓	✓	×	✓	○	✓	○	×	✓	✓	✓	×	✓	✓	✓	○	✓	○	✓	✓	✓	✓	18	78.2
**Leroux, Eric *et al***. [21]	✓	✓	✓	×	×	○	○	○	○	✓	✓	✓	×	✓	○	✓	✓	✓	○	✓	✓	✓	✓	17	74
**Dias, Amândio *et al***. [22]	✓	✓	✓	✓	✓	✓	✓	✓	○	×	✓	✓	✓	✓	✓	○	×	✓	○	✓	✓	✓	✓	19.5	84.7
**März, Karoline *et al***. [7]	✓	✓	✓	×	✓	○	✓	○	×	✓	✓	○	×	✓	✓	✓	○	✓	○	✓	✓	✓	✓	17.5	76
**Iacob, Simona Maria *et al***. [8]	✓	✓	✓	✓	✓	✓	✓	✓	✓	✓	✓	✓	×	✓	×	○	×	✓	○	✓	×	✓	✓	18	78.2
**Parrini, Simone *et al***. [13]	✓	✓	✓	✓	✓	✓	✓	○	○	✓	✓	✓	✓	✓	✓	✓	×	✓	✓	✓	✓	✓	✓	21	91.3
**Maurer, C. *et al***. [23]	✓	✓	○	×	✓	○	✓	○	×	✓	✓	○	×	✓	✓	○	○	✓	○	✓	✓	✓	✓	16.5	71.7
**Amaricai, Elena *et al***. [24]	✓	✓	✓	×	✓	○	✓	○	○	✓	✓	✓	×	✓	○	✓	×	✓	○	✓	✓	✓	✓	17.5	76.1
**Maurer-Grubinger, C. *et al***. [25]	✓	✓	✓	×	✓	○	✓	○	○	✓	✓	✓	×	✓	✓	✓	○	✓	○	✓	✓	✓	✓	18.5	80.4
**Bracco, P. *et al***. [26]	✓	○	✓	×	✓	○	✓	○	○	✓	✓	✓	×	✓	○	✓	×	✓	○	✓	✓	✓	✓	17	74
**Michalakis, Konstantinos X. *et al***. [27]	✓	✓	✓	×	✓	○	✓	○	✓	✓	✓	✓	×	✓	○	✓	×	✓	○	✓	×	✓	✓	17	74
**Tardieu, Corinne *et al***. [28]	✓	✓	✓	×	✓	○	○	○	○	✓	✓	✓	×	✓	✓	✓	✓	✓	○	✓	✓	✓	✓	18.5	80.4
**Michelotti, Ambrosina *et al***. [29]	✓	✓	✓	×	✓	○	✓	○	○	✓	✓	✓	×	✓	✓	✓	✓	✓	○	✓	✓	✓	✓	19	82.6
**Tecco, Simona *et al***. [30]	✓	✓	✓	×	○	○	○	○	○	×	✓	✓	×	✓	○	✓	×	✓	○	✓	✓	✓	✓	15.5	67.4
**Eriksson, Per Olof *et al***. [31]	✓	✓	✓	×	✓	○	✓	○	○	✓	✓	✓	×	○	✓	○	✓	✓	○	✓	✓	✓	✓	18	78.2
**Dias, Amândio A. *et al***. [32]	✓	✓	✓	○	×	○	×	○	○	×	✓	✓	○	✓	○	✓	×	✓	○	✓	✓	✓	✓	15.5	67.4
**Grondin, Francis *et al***. [33]	✓	✓	✓	✓	✓	✓	✓	✓	✓	✓	✓	✓	✓	✓	○	○	✓	✓	○	✓	×	✓	✓	20.5	89.1
**Nota, Alessandro *et al***. [4]	✓	✓	✓	✓	✓	✓	✓	✓	✓	✓	✓	✓	✓	✓	✓	✓	✓	✓	○	✓	✓	✓	✓	22.5	97.8

✓ denotes satisfactory fulfillment of methodological criteria (1.0 checkpoint); ○ represents partial fulfillment of methodological criteria (0.5 checkpoints); × signifies the absence of methodological criteria fulfillment (0.0 checkpoint); A–W refers to the criteria used in the methodological scoring presented in Table 2.

## Results

Regarding the type of studies analyzed in this systematic review, 13 (50%) studies were case series, 11 (42.3%) were cross-sectional studies, and 2 (7.7%) were cohort studies. Table 2 presents the methodological assessment tool used adapted from Lagravere *et al*. (2005), with modifications based on Bialocerkowski *et al*. (2010) [34]. The summary of the scores for the selected articles can be found in Table 3.

The average number of patients involved in the selected studies was 40, with some studies having much larger groups of 800 and 168 subjects. However, some studies evaluated only 15, 10, or even 7 patients. Seven studies had an experimental group and a control group; in one study, the experimental group subjects were also subjects in the control group. One study divided patients by age, and another divided patients into three categories: controls, patients with temporomandibular disorders, and patients with unilateral crossbite occlusion.

In 20 studies, the testing procedure took place over one day; in one study, the testing lasted two days with a break of 48 hours, and in another study, the testing lasted 3 days with breaks of 72 hours, the first days of testing serving as familiarization. Two studies had testing procedures that lasted one month with repositioning occlusal splints applied, and patients were tested every 7, 14, 21, and 28 days. Another study had a testing period of 6 months, with the splints' effect assessed every 3 and 6 months. One study assessed postural parameters before and after orthognathic surgery at 2.5 months.

### Risk of bias within studies

The equipment and subjects are well presented in all the studies, but the assessors were described only in 6 articles. Since the results on this topic have been controversial throughout the literature [22], it is recommended that observers with both dental and posture skills be involved in the design of interdisciplinary studies.

## Discussion

The measurement of postural parameters was primarily carried out using stabilometry platforms in most studies analyzed (17 studies) [4,5,27–30,32,35,6,16–18,20,21,24,26]. Other techniques included photogrammetry (6 studies) [7,13,17,22,25,31] combined with different techniques for three-dimensional spine reconstruction. In addition, two studies used mobile phone applications [8,33] that utilized a clinometer to calculate values obtained during body movement and an application that automatically traced and calculated body landmark deviations on frontal and profile photographs of subjects. Another study used the Fukuda-Unterberger test to assess vestibulospinal reflexes [15].

Although stabilometry platforms were the primary tools to analyze posture in most studies, we can observe in Table 1 that there is a wide variety of devices from different producers. In addition, the protocols regarding the period between measurements, the methods applied, and blinding measurements vary and may lead to dissimilar conclusions regarding the topic. Furthermore, stabilometry reduces all human postures to a single point, the center of foot pressure, which is assumed to be the gravity center, which may not provide a comprehensive understanding of posture behavior [26]. To address this limitation and highlight the importance of different sensorial inputs contributing to maintaining posture, we consider it worth measuring muscle tone alongside cognitive evaluation processes and vestibular feedback.

The test conditions varied among the studies, with 22 studies evaluating postural parameters in an upright position and static conditions [4,7,27–29,31–33,8,13,21–26] and 5 studies in moving conditions [6,23,28,30,35]. Movement conditions consisted of walking on a baropodometer, maintaining balance on an unstable platform with varying degrees of inclination, walking and running on a treadmill, or taking several steps with hands outstretched to check the maze function (Fukuda-Unterberger test) [15]. The occlusal conditions in which the postural measurements were performed were: interposition of cotton rolls between arches of different thicknesses, placed distal to the canines, uni and bilateral (12 studies) [4,5,30,35,7,16,20,23–26,29], maximum intercuspidation (19 studies) [4,5,24–28,30,33,35,36, 6–8,13,16–18,23], mandibular rest position (13 studies) [4,7,32, 33,35,13,17,18,20,24,27,28,31], silicone splints that modify the vertical dimension of occlusion [13,21,22,26,31,32], laterotrusion (3 studies) [7,8,27], two silicon panels (Flexi-Strips; Bausch KG, Cologne, Germany) of 2 mm thickness (2 studies) [6,23], bite a hard wax (Moyco) (1 study) [3], Impregum foils (1 study) [7].

Regarding the connections between the visual system and posture described in the literature, 4 studies took into account the closed-eyes open test [18,20,28,31]. The negative modification of these parameters means, in fact, a decrease in their values, so a postural imbalance [30]. Placing cotton rolls bilaterally without clenching leads to an increase in mean load pressure and a decrease in the total surface of the feet on the baropodometric platform, resulting in better stability when voluntary teeth clenching occurs [35]. However, placing the cotton rolls between the two arches may reduce the precision of proprioceptive periodontal information and induce different neck and cervical muscle activity due to trigeminal afferences [5]. Another reason for placing cotton rolls between the dental arches is that it can simulate a pathological condition of the stomatognathic system [19]. Other research found that using 4 mm thick occlusal devices on upper palatal incisors led to lower postural parameter values, including less body sway, slower sway speed, and a smaller sway movement area [31]. Lower angle of rotation values, representing deviations from the walking line, were observed in subjects wearing occlusal splints designed to evenly distribute occlusal forces, reduce muscle spasms and contractions, and eliminate lateral movement interferences [15]. The conflicting conclusions among studies may stem from several factors. In healthy individuals, teeth make contact for a limited duration (e.g., during chewing and swallowing activities), typically not exceeding 20-30 minutes per day [37], and the threshold sensitivity of periodontal ligaments is relatively low [38]. Introducing cotton rolls between teeth may not accurately simulate a TMD. The stimulation of trigeminal afferents could vary depending on the force exerted by each subject. Approximately half of the periodontal mechanoreceptors respond to the loading of groups of adjacent teeth, usually two to four teeth. The trigeminal influence on body muscle tone should be assessed individually since periodontal ligament receptors display a notably curved relationship between discharge rate and force amplitude, with the highest sensitivity to changes in tooth load at very low force levels (below 1 N for anterior teeth and 4 N for posterior teeth) [38]. Moreover, numerous histological studies emphasize the significance of well-developed mechanoreceptive innervation in the anterior part of the mouth [38–40]. Investigating the impact of low force levels of stimulation against anterior teeth on body posture would be of considerable interest.

The most common parameters used for posture analysis included oscillation area (7 studies), oscillation speed (8 studies), center of pressure position in the mediolateral and anteroposterior plane (6 studies), average distance of the center of pressure (3 studies), plantar pressure distribution (3 studies), postural stability index (2 studies), and spectral density (2 studies). In studies employing photogrammetric techniques to identify deviations in the sagittal or frontal plane, authors analyzed specific anatomical landmarks such as upper thorax inclination, spine outline, scapular belt, and posterior and anterior iliac spines.

## Conclusion

The stabilometry platform is the most frequently used tool for measuring postural parameters. However, other researchers have employed photogrammetry, mobile phone apps, and the Fukuda-Unterberger test to assess vestibulospinal reflexes. While some studies found no connection between occlusal changes and posture, 17 studies concluded that alterations in occlusal conditions could affect posture. Both orthognathic surgery and orthodontic braces significantly improve postural balance and athletic performance. Additionally, there are postural differences between Angle occlusal classes, with Angle classes II and III causing changes in postural values. Some studies also suggest that dental occlusion may influence posture, but only under specific movement, fatigue, and visual deprivation conditions. Occlusal devices, braces, silicone plates, and mouthguards, designed to simulate a malocclusion, impact the patients' postural system response.
